# Convolutional Autoencoder-Based Flaw Detection for Steel Wire Ropes

**DOI:** 10.3390/s20226612

**Published:** 2020-11-18

**Authors:** Guoyong Zhang, Zhaohui Tang, Jin Zhang, Weihua Gui

**Affiliations:** 1 School of Computer Science and Engineering, Central South University, Changsha 410083, China; csu_zgy@csu.edu.cn; 2 School of Automation, Central South University, Changsha 410083, China; zhtang@csu.edu.cn (Z.T.); gwh@csu.edu.cn (W.G.)

**Keywords:** flaw detection, wire rope, autoencoder, isolation forest, few training data

## Abstract

Visual perception-based methods are a promising means of capturing the surface damage state of wire ropes and hence provide a potential way to monitor the condition of wire ropes. Previous methods mainly concentrated on the handcrafted feature-based flaw representation, and a classifier was constructed to realize fault recognition. However, appearances of outdoor wire ropes are seriously affected by noises like lubricating oil, dust, and light. In addition, in real applications, it is difficult to prepare a sufficient amount of flaw data to train a fault classifier. In the context of these issues, this study proposes a new flaw detection method based on the convolutional denoising autoencoder (CDAE) and Isolation Forest (iForest). CDAE is first trained by using an image reconstruction loss. Then, it is finetuned to minimize a cost function that penalizes the iForest-based flaw score difference between normal data and flaw data. Real hauling rope images of mine cableways were used to test the effectiveness and advantages of the newly developed method. Comparisons of various methods showed the CDAE-iForest method performed better in discriminative feature learning and flaw isolation with a small amount of flaw training data.

## 1. Introduction

Flaw detection is an important and challenging task in many disciplines, such as hot strip mill rolling [1], the maintenance of bearings in rotary machinery systems [2,3], minerals processing [4], and the maintenance of steel wire ropes (SWRs) [5]. In the present paper, a case in point is the steel wire rope, which is widely used in lifting, transportation, and traction systems. Wire rope is composed of several strands of metal wire twisted into a helix forming a composite “rope”. Wire breakage will largely reduce the safety of wire ropes. At present, manual inspection under low-speed operation is still the main method used in most cases, which causes problems such as missed detection and false detection [6]. Therefore, progressive efforts have been devoted to exploring various methods to detect wire rope damages (e.g., computer vision method, electromagnetic method, ultrasonic guided wave method, X-ray detection method, etc.) in order to guarantee the reliability and safety of wire ropes [5]. It is known that due to normal friction and abnormal scratch, the early defects mainly occur on the wire rope surface, which often reflects as wear and broken wire damage [7]. In this work, efforts are devoted to developing a computer vision method to replace or assist the current manual inspection method to a certain extent because it can intuitively grasp the surface morphology characteristics of wire rope [7].

Extracting image features and identifying feature patterns are two major steps of computer vision-based abnormal detection [4]. As to the first step, principal component analysis (PCA) is a widely used feature extraction method [8]. It separates image data into principal component parts and residual parts. The principal component parts explain the highest amount of variance of the dataset, while the residual parts are less informative [9]. Zhou et al. [5] used the uniform local binary patterns (LBP) to extract rope texture features. LBP labels the pixels of an image by thresholding the neighborhood of each pixel and considers the result as a binary number. It is invariant to monotonic grayscale changes. In their later work [7], to make full use of the surface texture information of steel wire rope, a multi-texture feature fusion based on LBP and gray-level co-occurrence matrix (GLCM) was designed. To detect rope deformations, Vallan and Molinari [10] developed an automatic lay length measurement method, which extracts the rope contour and measures the distance among consecutive strands. However, the aforementioned feature extraction methods do not have learning ability. They are not powerful enough to extract important features from abnormal images, which results in poor fault detection performance [11,12]. To address the problem mentioned above and achieve automated key features extraction, this paper tries to develop a deep learning feature extraction method. Due to it being hard to prepare a sufficient amount of representative flaw data in practical applications, the convolutional denoising autoencoders (CDAEs) that can be trained using an unsupervised learning method are adopted [12,13]. To fully explore and exploit its learning ability, besides minimizing the reconstruction error between source images and reconstructed images, based on the flaw score calculated by a flaw detection model, CDAE is also trained to increase the flaw score difference between normal data and flaw data.

In previous studies, various classification techniques, including k-nearest neighbor, feedforward neural network, and support vector machine (SVM), were used to identify feature patterns in wire rope fault detection [5]. It is known that the performance of these methods relies heavily on the availability of a representative set of training data [14,15]. However, in real wire rope service, the life cycle of the cable is long, and it is difficult to collect flaw data. Furthermore, once the cable is severely damaged, it is replaced immediately. In this setting, it is difficult to prepare a representative set of industry data to train a well-performed computer vision-based fault classifier. Instead of training a fault classifier, in this study, we propose to build an Isolation Forest (iForest) model, considering that it is well applicable to cases with an insufficient amount of fault training data [4,16,17]. Generally, abnormal detection is formulated as finding the outlier deviating from the majority of observations [18]. In iForest, faulty samples are detected as the instances that have short average path lengths on the isolation trees (iTrees). This is because it is easy for the anomaly to be partitioned earlier when iTree grows. To mention a few, Zhang et al. [4] developed a fault detector for abnormal working condition recognition in froth flotation processes, in which Gabor texture features were extracted and fed to an iForest model to isolate abnormal instances [19]. In a study by Zhu et al. [20], an ensemble model of iForest was designed for detecting traffic anomalies within dashcam videos. Specifically, multiple iForest models at different scales were constructed to keep consistent spatial anomaly results over different frames. To detect insurance fraud on the basis of selected numeric attributes and distinct combinations of values of selected nominal attributes, Stripling et al. [21] constructed an iForest model and ran an analysis that was performed conditionally on well-defined data partitions. To well utilize the learning ability of deep learning features, this study tries to investigate the feasibility of whether the anomaly score calculated by an iForest model can be used to guide the finetuning of deep learning feature extraction models.

In this paper, according to the above investigation, the main contributions are summarized as follows: (a) a new convolutional autoencoder-based flaw detection method that can work with minimal flaw training data is developed; (b) the flaw score calculated by iForest is used to guide the finetuning of the convolutional autoencoder; (c) to show that the newly developed method is promising for practical wire rope monitoring, an application on the flaw detection of the hauling rope of a mine cableway is conducted in a real mineral concentration plant.

The remainder of the paper is organized as follows: The problem of wire rope flaw detection is explained in Section 2. Section 3 proposes the flaw detection method. In Section 4, the proposed method is validated through experiments, some comparative studies are conducted, and some key issues are discussed in detail. Section 5 presents the conclusions of this study.

## 2. Wire Rope Image Representation

### 2.1. Preliminaries of Denoising Autoencoder

An autoencoder is a neural network containing an encoder and a decoder [22,23]. The encoder takes an input vector *x* and maps it to a latent representation *z* through a deterministic mapping $z=E_{\theta_{e}}\left(x\right)=\sigma \left(W_{e}x+b_{e}\right)$, parameterized by $\theta_{e}=\left\{W_{e},b_{e}\right\}$, while the decoder maps the latent representation *z* back to a “reconstructed” vector $\tilde{x}$ in input space $\tilde{x}=D_{\theta_{d}}\left(z\right)=\sigma \left(W_{d}x+b_{d}\right)$, parameterized by $\theta_{d}=\left\{W_{d},b_{d}\right\}$. $\theta_{e}$ and $\theta_{d}$ are optimized to minimize the average reconstruction error:

(1) $$\theta_{e}^{*},\theta_{d}^{*}=argmin\frac{1}{n}\underset{i}{\sum }\ell \left(x_{i},{\tilde{x}}_{i}\right)$$

where ℓ is a loss function, such as the traditional squared error $\ell \left(x,\tilde{x}\right)=∥x-\tilde{x}{∥}^{2}$. However, the criterion, which encourages latent representation *z* to retain information of input *x* alone, is unable to guarantee the extraction of discriminative image features. This is because this criterion can easily lead to the simple solution “copy the input” or similarly uninteresting ones that trivially maximize mutual information [24].

Vincent et al. [13,24] developed a denoising autoencoder, which is trained to reconstruct a clean “repaired” input from a corrupted version of it. Specifically, the initial input *x* is firstly corrupted into a partially destroyed version $\hat{x}$ by a stochastic mapping $\hat{x}∼q_{D}\left(\hat{x}|x\right)$. Then, the corrupted input $\hat{x}$ is mapped to a latent representation $z=E_{\theta_{e}}\left(\hat{x}\right)$. Next, the original input is reconstructed from z with $\tilde{x}=D_{\theta_{d}}\left(z\right)$. To encourage the similarity between $\tilde{x}$ and the uncorrupted input *x*, encoder and decoder parameters are trained to minimize the average reconstruction error in (1). Note that unlike the basic autoencoder, the deterministic mapping $E_{\theta_{e}}$ in the denoising autoencoder is applied to a corrupted input. It thus forces the learning of a far more clever mapping than the identity: one that extracts features useful for denoising [13].

### 2.2. Convolutional Denoising Autoencoder-Based Feature Extraction

The convolution denoising autoencoder combines the local convolution connection with the autoencoder. Specifically, convolutional operations are used to replace fully connected layers in encoders, while deconvolution operation is used to replace fully connected layers in decoders [25]. Training deep neural networks is complicated by the fact that the distribution of each layer’s inputs changes during training, as the parameters of the previous layers change [26]. To address the problem, in this study, instance normalization is introduced in both encoders and decoders.

- Convolution layer: The convolution layer employs the learnable filter to slide with a prescribed stride and outputs feature maps. Each element in a feature map is the sum of the products of the filter and the input image (or the previous feature map) with which this filter overlaps. Let the *k*-th feature map of the *l*-th layer be $O_{l}^{k}\in {\mathbb{R}}^{w_{l}\times h_{l}}$, and the corresponding filter be $w^{k}\in {\mathbb{R}}^{w_{l}^{\prime }\times h_{l}^{\prime }}$. $O_{l}^{k}$ is calculated as follows:
  (2) $$\begin{array}{l} O_{l}^{k}\left(i,j\right)=\sigma \left(\overset{d_{l-1}}{\underset{k^{\prime }=1}{\sum }}w_{k^{\prime }}^{k}\left(i,j\right)*O_{l-1}^{k^{\prime }}\left(i,j\right)+b_{k}\right)\\ =\sigma \left(\overset{d_{l-1}}{\underset{k^{\prime }=1}{\sum }}\overset{\frac{w_{l}^{\prime }}{2}}{\underset{\tau_{1}=-\frac{w_{l}^{\prime }}{2}}{\sum }}\overset{\frac{h_{l}^{\prime }}{2}}{\underset{\tau_{1}=-\frac{h_{l}^{\prime }}{2}}{\sum }}w_{k^{\prime }}^{k}\left(\tau_{1},\tau_{2}\right)O_{l-1}^{k^{\prime }}\left(i-\tau_{1},j-\tau_{2}\right)+b_{k}\right) \end{array}$$
  where $d_{l-1}$ is the number of channels in layer *l* − 1; *i* and *j* are the row and column indices, respectively; $b_{k}$ is a bias; and σ is an activation function. A Leaky ReLU with a negative slope of 0.2 is used in this study. The parameters related to the convolutional operation are the filter size, the stride, and the number of feature maps.
- Deconvolution layer: The deconvolutional layer also employs filters to slide with a prescribed stride. Similar to Equation (2), each element in a feature map is the sum of the products of the filter and the previous feature maps with which this filter overlaps. Different from convolutional layers, ReLU is used as the activation function. In addition, in order to increase the resolution of its input, extra zeros are padded to the input feature maps.
- Instance Normalization: Let $x\in {\mathbb{R}}^{T\times C\times W\times H}$ be an input tensor containing a batch of *T* images. Let $x_{t,k}^{i,j}$ represent the *tkij*-th element, where *t* is the index of the *t*-th image in the batch, *k* is the channel index, and *i* and *j* are the row and column indices, respectively.
  (3) $$\begin{array}{l} y_{t,k}^{i,j}=\frac{x_{t,k}^{i,j}-\mu_{t,k}}{\sqrt{\sigma_{t,k}^{2}+\epsilon }}\\ \mu_{t,k}=\frac{1}{HW}\overset{W}{\underset{l=1}{\sum }}\overset{H}{\underset{m=1}{\sum }}x_{t,k}^{l,m}\\ \sigma_{t,k}^{2}=\frac{1}{HW}\overset{W}{\underset{l=1}{\sum }}\overset{H}{\underset{m=1}{\sum }}{\left(x_{t,k}^{l,m}-m\mu_{t,k}\right)}^{2} \end{array}$$

**Figure 1:**
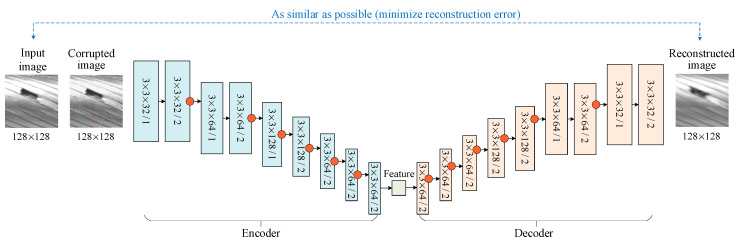
The architecture of the convolutional autoencoder. Red circles represent that instance normalization is used after convolution.

The architecture of the convolutional denoising autoencoder designed in this study is illustrated in Figure 1. The encoder contains nine convolutional layers, and correspondingly, nine deconvolutional layers are used in the decoder. Since it is better to use a larger receptive field in lower convolutional layers, the first four convolutional layers in the encoder are grouped into two groups. A stride of one is used in the first convolutional layer in each group, and a stride of two that aims to achieve subsampling is used in the second convolutional layer. There are no pooling layers; down-sampling in remaining layers is also implemented with a stride of two. Instance normalization is used after each down-sampling convolutional (up-sampling deconvolutional) layer.

Because it is hard to train the convolutional denoising autoencoder from randomly initialized parameters, the greedy layer-wise procedure is used to pre-train the encoder and the decoder layer-by-layer with a local reconstruction loss [13]. Specifically, convolutional layers in the encoder and the corresponding deconvolutional layers in the decoder are short-connected and pre-trained to minimize a reconstruction loss between the inputs of this convolutional layer and its corresponding outputs in the deconvolutional layers. After pre-training, convolutional layers and deconvolutional layers are stacked together as in Figure 1 and finetuned by minimizing the reconstruction loss between the input image and restored image.

## 3. Flaw Detection with Deep Learning Features

### 3.1. Isolation Forest-Based Flaw Detection

Isolation Forest (iForest) is an unsupervised, tree-based ensemble anomaly detection method [4,16,17]. The base learner of iForest is the isolation tree (iTree). There are two kinds of nodes in iTree: One is an external node that has no child. The other is an internal node that has one test and exactly two daughter nodes ($T_{l}$, $T_{r}$). An iTree node is created by randomly selecting an attribute along with a randomly drawn split value. The split value lies between the minimum and maximum of the selected attribute. In other words, a node consists of a randomly chosen attribute *q* and a randomly drawn split value *p*, such that the attribute *q* divides the data points into $T_{l}$ and $T_{r}$. Usually, an iTree is built as follows. Given a training set $X={\left\{x_{i}\right\}}_{i=1}^{N},x_{i}\in {\mathbb{R}}^{m}$, a subset (whose size is ψ) of data are taken from the training data X without replacement. In a divide-and-conquer fashion, iTree recursively divides the input space into a progressively smaller subspace by randomly selecting an attribute *q* and a split value *p* until one of the following conditions holds: (i) the tree reaches a predefined height limit; (ii) |X|=1, or (iii) all data items in *X* have the same values. A detailed algorithm for constructing iTree is summarized in Algorithm 1.
**Algorithm 1.** Isolation tree construction.**Isolation tree:**iTree(X,e,l) **Inputs:***X*- input data, *e* - current tree height, *l* - height limit **Output:** an *iTree* 1:  **if**
e≥l or |X|≤1
**then** 2:   return exNode{Size←|X|} 3:  **else**
4:   let Q be a list of attributes in *X* 5:   randomly select an attribute q∈Q 6:   randomly draw a split value p from max and min value of q 7:   $X_{l}\leftarrow filter\left(X,q<p\right)$
8:   $X_{r}\leftarrow filter\left(X,q\geq p\right)$ 9:   return $inNode\{Left\leftarrow iTree\left(X_{l},e+1,l\right)$ 10:          $Right\leftarrow iTree\left(X_{r},e+1,l\right)$ 11:          SplitAtt←q 12:          SplitValue←p} 13:  **end if**

When a test instance passes through an iTree, to decide whether it should traverse to the left or right daughter node, its respective attribute value is retrieved and compared to the split value at each internal node. In iForest, it is assumed that anomalies or flaws only take a very small portion in the data space, and they are different from normal instances. Thus, flaws are more susceptible to be isolated in an iTree and tend to reach the terminated external node earlier than normal instances. Therefore, when a collection of iTrees collectively produces a shorter traverse path for some particular instances, then these instances are highly likely to be faulty. Let $h_{t}$ represent an iTree. The path length $h_{t}\left(x\right)$ of *x* traversing $h_{t}$ can be measured by the number of edges that *x* traverses from the root node to the terminated external node. To account for the possibility that the isolation of a set of instances at the external node did not fully succeed, an adjustment function based on the external node size $n_{ex}$ is added to the path length, as shown below:

(4) $$\begin{array}{c} h_{t}\left(x\right)=e+c\left(n_{ex}\right)\\ c\left(n_{exNode}\right)=\left\{ \begin{array}{l} 2H(n_{ex}-1)-\frac{\left(n_{ex}-1\right)}{n_{ex}}\;\mathrm{if}n_{ex}>2,\\ 1\;\mathrm{if}n_{ex}=2,\\ 0\;\mathrm{otherwise} \end{array}\right. \end{array}$$

where H(i) is the harmonic number, which can be estimated by ln(i)+0.5772156649 (the Euler’s constant). The flaw score of an instance is defined as follows [23]:

(5) $$s\left(x,\psi \right)=2^{-\frac{E\left(h\left(x\right)\right)}{c\left(N\right)}}$$

where E(h(x)) is the average of h(x) in a collection of iTrees.

- If the average path length of *x* is close to zero (E(h(x))→0), the flaw score will be close to one (s(x,ψ)→1), and hence *x* tends to be a flaw instance.
- If the average path length of *x* is close to the absolute maximum depth (E(h(x))→ψ−1), the flaw score will be close to zero (s(x,ψ)→0), and hence *x* tends to be a normal instance.
- If the average path length of *x* is close to the average path length of a random tree given *ψ* (E(h(x))→c(ψ)), the flaw score will be close to 0.5 (s(x,ψ)→0.5). Then, there are no distinct flaws in the data.

### 3.2. Flaw Score-Based CDAE Finetuning

The isolation method assumes that abnormal or flaw instances are different from normal instances in the feature space. However, CDAE is constructed with no supervised information. To augment the reliability of iForest and increase the difference gap of CDAE features between flaw images and normal images, in this study, the flaw score difference between normal data and flaw data is used to finetune the learnable parameters of CDAE. Hence, a novel penalty term that is calculated as follow is defined:

(6) $$\ell_{flaw}=\underset{i\in S_{F}}{\sum }s\left(x_{i}^{F},\psi \right)-\underset{i\in S_{N}}{\sum }s\left(x_{i}^{N},\psi \right)$$

where $x_{i}^{F}$ means a training data sampled from a randomly chosen flaw image subset, and similarly, $x_{i}^{N}$ means a training data sampled from a randomly chosen normal image subset, and *s* is an Euclidian distance-based similarity function.

The detailed steps of the flaw detection scheme based on CDAE and iForest are presented as follows:

Stage I: Offline construction
  Step 1: Get the sample set *X* under normal operating conditions and normalize it.
  Step 2: Initialize CDAE and pretrain its layers to be greedy to minimize a reconstruction loss between the inputs of the convolutional layer and its corresponding outputs in the deconvolutional layer.
  Step 3: Adjust the parameters of CDAE by minimizing the reconstruction loss between the input image and restored image.
  Step 4: Build the iForest model with CDAE features.
  Step 5: Finetune the parameters of CDAE to increase the gap between flaw scores of normal data and flaw data.
  Step 6: Calculate the flaw score of incoming data.

Stage II: Online monitoring
  Step 1: Capture a new data and normalize it.
  Step 2: Calculate the CDAE image features of the new data.
  Step 3: Calculate the flaw score by feeding CDAE features to iForest model.
  Step 4: Determine whether the input data is flaw data or normal data.
  Step 5: If it is a flaw data, trigger the flaw alarm; otherwise, capture the next data.

## 4. Case Study: Application of Hauling Rope Flaw Detection on Mine Cableway

### 4.1. Description of Hauling Rope Flaw Detection

**Figure 2:**
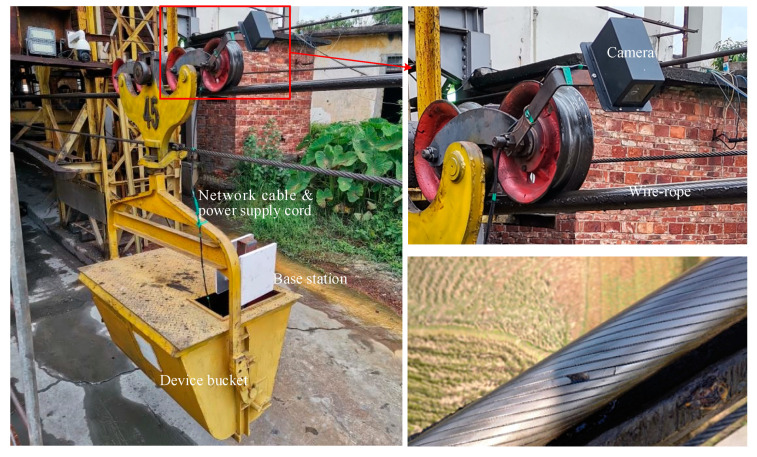
System structure of the hauling rope flaw detection in mine cableways.

The hauling rope of mine cableways is taken as an example in this paper. The wire rope system is composed of two different rope specifications, which are 50 mm and 32 mm in nominal diameter. The nominal length of the two types of ropes is 1500 m; the rope with a diameter of 50 mm is used to carry the bucket wagon with the mine, and the rope with a diameter of 30 mm is used to carry the empty wagon. The system structure is shown in Figure 2. The system data is stored in a Device Bucket, which contains industrial control computers, camera power supplies, Gigabit Ethernet switches, and UPS uninterruptible power supplies. The image acquisition part of the system consists of a GIGE protocol industrial camera named MV-CA030-10GC (Hikvision, Hangzhou, Zhejiang Province, China) with a resolution of 1920 × 1440 pixels (large portions of the captured images consist of backgrounds) and a bit depth of 8 Bit. The camera captures real-time image data at 20 frames per second, and the traveling speed of the truck is 0.2 m/s. In the wireless network, two signal base stations are responsible for communication with the two rope base stations to ensure that the system supports remote viewing of the rope’s real-time maintenance status.

**Figure 3:**
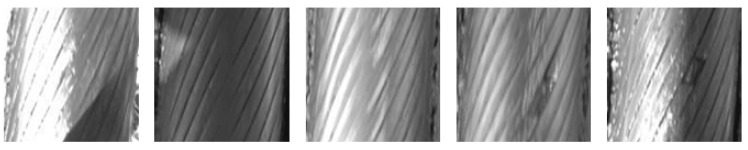
Real samples of wire rope images captured from hauling ropes.

To train and test the performance of our flaw detection method, 113 flaws were collected from nine hauling wire ropes that were to be replaced. For each hauling wire-rope, there were more than ten thousand images. Some samples are shown in Figure 3. Due to complex natural light conditions in an outdoor setting, there are many artifacts in captured images, as shown in the first two images. It can be observed from the third and the fourth image, the surface of the cable becomes smooth due to long-term wear and tear, which reduces the contrast of flaw regions. Also, the lubricating oil and dust will cause highlighted or darkened areas, which result in noises for flaw region detection. The camera aperture was dynamically adjusted to reduce the disturbance of light reflection on the wire rope surface when capture hauling rope images.

### 4.2. Evaluation Metrics

To measure the performance of the different flaw detection approaches, four evaluation metrics, named *accuracy*, *precision*, *recall*, and *f*1*-score*, are calculated. These metrics are calculated according to the following formulae, and detailed descriptions can be found in the literature [5,11].

(7) $$accuracy=\frac{TP+TN}{TP+FP+FN+TN}$$

(8) $$precision=\frac{TP}{TP+FP}$$

(9) $$recall=\frac{TP}{TP+FN}$$

(10) $$f1-score=2\frac{precision\times recall}{precision+recall}$$

where *TP* is true positive, *FP* is false positive, *TN* means true negative, and *FN* means false negative.

### 4.3. Experiments and Results

**Figure 4:**
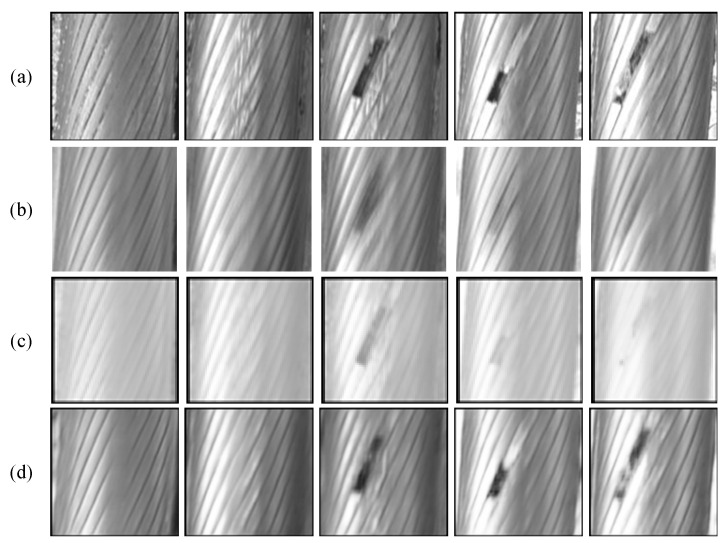
Comparison between the original wire rope images and their reconstructions. (**a**) The original wire rope images; (**b**–**d**) Images reconstructed by PCA, stacked autoencoder (SAE), and the proposed method, respectively.

The convolutional denoising autoencoder acts like a method of dimensionality reduction. Thus, in the first experiment, we compare it to two traditional dimensionality reduction methods: PCA and stacked autoencoder (SAE). In the experiment, the first 32 principal components are chosen to make a reconstruction. The encoder in SAE has five fully connected layers, and the output dimension of these layers is 2048, 1024, 256, 64, and 32, respectively. The input of the encoder is downsampled into 64 × 64. Reconstruction results of five randomly selected wire rope images are displayed in Figure 4. The reconstructions from the proposed method almost restored all normal and flaw parts of the original wire rope images. For example, gaps between any two strands of metal wires, which indicate connectivity of wire ropes, remain in the reconstruction of CDAE. By contrast, it is almost impossible to distinguish independent wire rope strands from SAE and PCA. In addition, PCA failed to restore flaw parts.

The signal-noise ratio (SNR) and root mean squared error (RMSE) are used to evaluate the reconstruction errors. Evaluation results for randomly selected 1000 wire rope images are summarized in Table 1. As can be seen, the proposed CDAE outperforms its competitors. The achieved RMSE is 18.6% and 57.3% lower than that of PCA and SAE, respectively, and this may indicate that the proposed method will perform better than its competitors in wire rope feature extraction. PCA assumes that the principal components with the highest variance contain the most discriminable information that allows us to separate images by their labels. Thus, it looks at the image set as a whole and determines the direction of the highest variance. Then it determines the next direction of highest variance, which is orthogonal to the previous ones, and so on. However, since there are few flaw data, the separation of flaw images and normal images is not based on the maximum variance. Thus, the use of the most important components of PCA will not work. The aim of SAE is to train an encoder and a decoder in such a way that only minimal latent information is required to restore the input image. Suppose we use too few nodes in the bottleneck layer: in that case, the capacity of SAE to restore the input image will be limited, and the synthesized result will be blurry or unrecognizable from the input image. Thus, the experiment results indicate that CDAE performs better than SAE by adopting convolutional operations.

**Figure 5:**
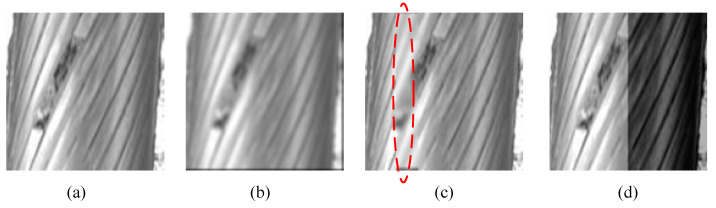
Flaw image augmentation. (**a**) Source images; (**b**–**d**) Image augmentation by applying Gaussian smoothing, reducing local contrast, and adding shadow, respectively.

The second experiment studies the flaw detection performance of the proposed method. To illustrate its advantages, it is compared with existing representative works in this field, as summarized in Table 2. Algorithm I [8] uses PCA to extract principal features associated with wire rope features. Then, an SVM model that trained with the one class-based multi-class optimization algorithm is used to identify flaw images. In Algorithm II [7], a mixture of LBP and GLCM is used to represent wire rope images. Then, an SVM model optimized with an improved fruit fly optimization algorithm is used in flaw identification. In Algorithm III, image encoding results of SAE are used as the representation of input images, and an SVM-based classifier is used as the flaw identification model. To show the advantage of Isolation Forest-based flaw identification in the situation that only a limited size of flaw data is available, a comparison model, which integrates convolutional denoising autoencoder-based feature representation and SVM-based flaw identification, is constructed and denoted as Algorithm IV. At the same time, since convolutional layers are used in CDAE, to compare the performance between CDAE and convolutional neural networks (CNNs), a CNN developed in [27] is reimplemented and used to extract deep learning features for iForest. In the experiment, we name this CNN and iForest-based method as Algorithm V. As aforementioned, there are only 113 independent flaw images in the collected hauling rope data. As shown in Figure 5, to train an SVM classifier, each flaw image is copied three times to augment the data set by adopting image operations like applying Gaussian smoothing, reducing local contrast, and adding shadow. Thus, there are a total of 452 flaw images. Additionally, 5100 normal images are randomly selected from collected data. To train and test the above fault detection models, 100 flaw images and 100 normal images are randomly selected as test samples. The remaining data are used as training data. Due to limited flaw data, the above four flaw detection models were cross-validated several times (i.e., the training sets and test sets were divided repeatedly). The model that gave the best performance (least error) was selected as the best-trained detection model.

The experiment results are summarized in Table 2. It can be observed that the performance of Algorithms V and II are similar. The poor performance of Algorithm V is mainly caused by the limited amount of flaw training data. Algorithm IV performs better than Algorithms I and III, which use PCA and SAE features, respectively. Due to the poor performance of PCA on flaw region characterization (as shown in Figure 4), it is hard for PCA features-based Algorithm I to discriminate flaw images from normal images. Though SAE performs slightly better than PCA on flaw region characterization, it still has difficulties in detecting small flaws, and hence results in poor performance in small flaw detection. Due to limited flaw training data, the proposed fault detection method that combines CDAE with iForest has higher precision than SVM classifier-based Algorithm IV.

## 5. Conclusions

In this study, a new wire rope flaw detection method that combines convolutional denoising autoencoder (CDAE) and isolation forest (iForest) was developed. Different from previous work, the newly developed method has learning ability and can perform well with a small amount of flaw training data. It is known that iForest is based on the assumption that abnormal instances are largely different from normal instances. In this study, a novel penalty term, which was calculated as the flaw score difference between normal data and flaw data, was defined to adjust the learnable parameters of CDAE to encourage iForest to separate flaw data with shorter path lengths. The advantages of the proposed method were verified by comparing it with four other visual perception-based fault identification methods. First, image reconstruction experiments demonstrated that the new method could capture the flaw appearance and structure characteristics. Second, comparative experiments involving real-world hauling rope flaw identification provided evidence that the new method performs better than its competitors with few flaw training data.

## Figures and Tables

**Table 1:** Quantitative evaluation of wire rope image reconstruction. SNR: signal-noise ratio; RMSE: root mean squared error.

	Method		
	PCA	SAE	Proposed
SNR	6.5656	1.0592	8.4465
RMSE	7.3944	14.1201	6.0218

**Table 2:** Temperature and wildlife count in the three areas covered by the study. LBP: local binary patterns; GLCM: gray-level co-occurrence matrix; CDAE: convolutional denoising autoencoder; CNN: convolutional neural network.

Method	Feature	Isolation Model	Metric			
			Accuracy	Precision	Recall	F1-Score
Algorithm I [8]	PCA	SVM	0.775	0.784	0.76	0.772
Algorithm II [7]	LBP + GLCM	SVM	0.805	0.814	0.79	0.802
Algorithm III	SAE	SVM	0.865	0.884	0.84	0.861
Algorithm IV	CDAE	SVM	0.885	0.905	0.86	0.882
Algorithm V	CNN	iForest	0.835	0.876	0.78	0.823
Proposed	CDAE	iForest	0.93	0.948	0.91	0.929
